# Evaluating depression- and anxiety-like behaviors in non-human primates

**DOI:** 10.3389/fnbeh.2022.1006065

**Published:** 2023-01-19

**Authors:** Karla K. Ausderau, Ricki J. Colman, Sabrina Kabakov, Nancy Schultz-Darken, Marina E. Emborg

**Affiliations:** ^1^Wisconsin National Primate Research Center, University of Wisconsin—Madison, Madison, WI, United States; ^2^Waisman Center, University of Wisconsin—Madison, Madison, WI, United States; ^3^Department of Kinesiology, University of Wisconsin—Madison, Madison, WI, United States; ^4^Department of Cell and Regenerative Biology, University of Wisconsin—Madison, Madison, WI, United States; ^5^Department of Medical Physics, University of Wisconsin—Madison, Madison, WI, United States

**Keywords:** depression, anxiety, non-human primates, macaque, marmoset

## Abstract

Depression and anxiety are some of the most prevalent and debilitating mental health conditions in humans. They can present on their own or as co-morbidities with other disorders. Like humans, non-human primates (NHPs) can develop depression- and anxiety-like signs. Here, we first define human depression and anxiety, examine equivalent species-specific behaviors in NHPs, and consider models and current methods to identify and evaluate these behaviors. We also discuss knowledge gaps, as well as the importance of evaluating the co-occurrence of depression- and anxiety-like behaviors in animal models of human disease. Lastly, we consider ethical challenges in depression and anxiety research on NHPs in order to ultimately advance the understanding and the personalized treatment of these disorders.

## 1 Introduction

Depression is a common mental health disorder that often presents with anxiety. Both mental conditions are frequent comorbidities of a range of diseases, including diabetes, Parkinson’s disease (PD), and asthma. The World Health Organization estimated that more than 280 million people of all ages from around the world suffer from depression and 301 million live with an anxiety disorder (Institute of Health Metrics and Evaluation, 2022). Although the etiologies of both depression and anxiety are not well understood, their association with dysfunction of brain regions within the limbic system or “emotional” brain and the reward system neural network is well documented (Milak et al., 2005; Malhi and Mann, 2018).

Nonhuman primates (NHPs) share genetic, physiological, neuroanatomical, and behavioral attributes with humans that make them valuable for modeling human diseases, including depression and anxiety (Birn et al., 2014; Pessoa and Hof, 2015). NHPs are outbred like humans and thus have a similar degree of inter-individual variability. They undergo developmental stages similar to humans, and their relatively long lifespan compared to rodents aids in the evaluation of changes across the lifespan (Capitanio and Emborg, 2008; Ausderau et al., 2017). Like humans, NHPs (but not other nonhuman species) have 3 unique characteristics of the limbic system: a reduced olfactory lobe and bulb, a cingulate gyrus with greater size in the caudal regions, and an enlarged frontal lobe (Pessoa and Hof, 2015; van Heukelum et al., 2020).

It is difficult to know whether an animal is truly depressed or anxious, as they cannot express with words feelings of hopelessness or distractibility. Yet, NHPs can present depression- and anxiety-like behaviors (Table 1) that can provide insight into the human condition (Bliss-Moreau and Rudebeck, 2021). For example, studies in macaques with the endophenotype [heritable traits derived from laboratory measures (Iacono, 2018)] for “anxious temperament” have detected inheritable patterns of metabolic activity in the hippocampus that were predictors of anxious-like behaviors later in life (Oler et al., 2010). In order to guide clinical and preclinical studies in mental health disorders, the US National Institute of Mental Health developed the Research Domain Criteria (RDoC) that include six psychological domains: cognitive systems, arousal and regulatory systems, social processes, sensorimotor systems, and negative valence systems. Each domain has seven units of analysis: molecules, cells, circuits, physiology, behavior, self-report, and paradigms (RDoC Matrix, 2022). This matrix provides a framework to analyze the data collected in animal models, the presence of a relevant endophenotype, and their overall value for clinical translation. Critical for studies on depression and anxiety, it exposes that, of the six RDoC, the self-report domain cannot be studied in the NHP model (French, 2019; Gururajan et al., 2019).

**Table 1:** Human signs and symptoms of depression and anxiety and their equivalent non-human primate (NHP) depression- and anxiety-like signs.

**Depression and Anxiety Signs and Symptoms**		
Human^#^ 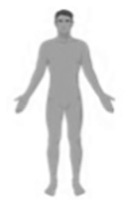	Macaque 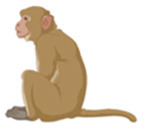	Marmosets 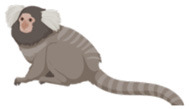
Lack of motivation/lack of interest*	Lack of interest in preferred foods, lack of interest in novel objects	Lack of interest in preferred foods, lack of interest in novel objects
Loss of interest in others*	Avoids others in the group, withdrawal from any approach by others, sits alone, no active social interactions	Avoids others in group, withdrawal from any approach by others, sits alone, no active social interactions
Reports of sadness*	Increased “hoo” vocalizations when iso-lated from group, lack of interest in others’ activity	Increased “phee” vocalizations when isolated from group, lack of interest in all activity
Apparent sadness in expression/posture*	Sits in self-clasping huddle position with hunched posture	Sits in hunched posture with head down, sometime with messed fur
Concentration difficulties	Difficulties with problem solving, trouble following learned tasks	Difficulties with problem solving, trouble following learned tasks
Slow movement or agitated movements	Long periods of passive sitting or rapid pacing and/or hopping	Long periods of passive sitting or constant vigilant behavior with alarm “tse” vocalizations
Agitation**	Movement stereotypies (i.e., circling in place, hair pulling, saluting, self-biting)	Constant vigilant jumping from branch to branch accompanied by “tse” vocalizations
Fear**	Vocalizations, vigilance, freezing posture	Hides in back of cage away from others
Loss of appetite	Not eating during regular feedings and ignores treats	Not eating during regular feedings and ignores treats
Sleep disturbances	Activity during sleep phase, or sleeping during day	Sleeping during times of group activity
Somatic symptoms (e.g., pain, difficulty breathing, gastrointestinal dysfunction)	Somatic signs (e.g., diarrhea, constipation, increased heart rate, altered breathing)	Somatic signs (e.g., diarrhea, increased heart rate, altered breathing, messed fur)

^#^American Psychiatric Association (2013). It should be noted that although freezing and vocalization behaviors are adaptive in the defensive repertoire of NHPs, the characteristics of the response (e.g., intensity, duration) in NHPs exhibiting depression- and anxiety-like behaviors differs (e.g., : intensity, duration) from a normal response (Fox and Kalin, 2014). *corresponds to typical signs of depression; **corresponds to typical signs of anxiety. See Tables s 2–4 for detailed marmoset and rhesus specific behavior evaluations and associated citations. Icons were created with BioRender.com.

Advances in identification of alleles associated with human disorders as well as in methods for genomic editing is envisioned as an opportunity to generate NHP disease models based on germline (Aida and Feng, 2020) or somatic cell genomic modification (Saha et al., 2021). These new models are expected to genocopy and phenocopy disorders that may develop depression or anxiety as independent entities or as comorbidities. Here we aim to reflect on human depression and anxiety and equivalent behaviors in NHPs, in order to ethically capitalize on emerging models and methods of evaluation to ultimately advance the understanding and treatment of these disorders.

## 2 Depression and Anxiety in Humans

### 2.1 Human depression and anxiety are complex disorders

Depression is defined as extended periods of a sad mood that interferes with everyday functioning (Brody et al., 2018). It is typically diagnosed by a medical professional through a diagnostic evaluation using the Diagnostic and Statistical Manual of Mental Disorders (DSM-5) criteria (American Psychiatric Association, 2013). The prevalence of depression is difficult to estimate as it can occur across the lifespan, its manifestations are heterogeneous and vary by age, sex, income, and health behaviors (Villarroel and Terlizzi, 2020). Although mental, social, and physical symptoms of depression present with individual differences, over 80% have difficulty with their daily activities due to these symptoms (Brody et al., 2018). Different types of depression vary in the range of severity and length of symptomology (Table 1). For example, major depressive disorder is defined by severe symptomology that occurs most of the time compared to milder forms of depression, such as persistent depressive disorder that is a chronic state of milder symptomology but can be equally as debilitating. Depressive symptoms are also a key component of other disorders such as psychotic depression and bipolar disorder and, in addition, they may occur under unique circumstances, like postpartum depression or seasonal affective disorder.

Anxiety disorders are a common mental health condition that typically persists for months if not years (Anxiety Disorders, 2022). Individuals with anxiety disorders often struggle with intense and uncontrollable feelings of anxiety, fear, worry, and/or panic (American Psychiatric Association, 2013). Anxiety disorders include a number of related disorders including generalized anxiety disorder, panic disorder, social anxiety disorder, and various phobia-related disorders (American Psychiatric Association, 2013). While unique in their clinical presentation, the symptoms across disorders are persistent over time and interfere with an individual’s ability to participate in daily activities such as social relationships, work performance, and community engagement (Anxiety Disorders, 2022).

Other mental health conditions often co-occur with depression, including generalized anxiety disorders (Hirschfeld, 2001; Kessler et al., 2008). Shared environmental and genetic risk factors are suggested to contribute to the high rates of co-occurrence (50%) and shared symptomology of depression and anxiety disorders (Wittchen et al., 2000; Pollack, 2005; Lacerda-Pinheiro et al., 2014). Together they can have a cascading impact on an individual’s daily functioning, including self-care, social engagement, and occupational performance (Hirschfeld, 2001; Read et al., 2017).

### 2.2 Depression and anxiety can be comorbidities

Depression and anxiety can present independently and as comorbidities or as part of different disease processes. Patients with prevalent chronic disorders such as diabetes, arthritis, asthma, chronic obstructive pulmonary disease, stroke, and PD frequently have depression and/or anxiety that affect the primary illness management and prognosis (Reijnders et al., 2008; Morimoto et al., 2015; Zhang et al., 2017). In fact, people with chronic physical conditions or multiple chronic conditions are 2–3 times more likely to be diagnosed with depression (Read et al., 2017). Due to high rates of co-occurrence and overlap of symptomatology, depression often goes undiagnosed when associated with chronic disease (Morimoto et al., 2015; Mourao et al., 2016; Ismail et al., 2017). Anxiety is hypothesized to be even more prevalent than depression as co-occurring with physical illness and equally undiagnosed and inappropriately treated (Sartorius et al., 2014; Meuret et al., 2020). A strong connection has been established between anxiety disorders and some gastrointestinal diagnoses with up to 95% of patients reporting anxiety in certain conditions, such as irritable bowel syndrome (Whitehead et al., 2002; Härter et al., 2003; Fadgyas-Stanculete et al., 2014). Co-occurrence with chronic pain disorders such as fibromyalgia is also particularly high (Csupak et al., 2018; Montesó-Curto et al., 2018). Accurate diagnosis and treatment of both disorders is essential due to the high economic burden, and the need to guide appropriate clinical decisions and public health policy.

### 2.3 Current treatments for depression and anxiety have limitations

Depression is considered to be a chronic but treatable mental disorder with responses ranging from full recovery to ongoing treatment needed. Management plans typically consist of psychotherapy and or medications (e.g., selective serotonin reuptake inhibitors, SSRIs; Cuijpers et al., 2013; Brody and Gu, 2020). Lifestyle changes (e.g., physical activity and mindfulness-based interventions) are now also often recommended as part of the therapeutic approach (Khoury et al., 2013; Rosenbaum et al., 2014; Blanck et al., 2018). Approximately 10%–20% of individuals do not respond to treatment over time (Kessler et al., 2005; Center for Behavioral Health Statistics and Quality, 2018). For treatment-resistant cases, focal neuromodulation (e.g., vagus nerve stimulation, deep brain stimulation, transcranial magnetic stimulation) or, brain stimulation therapies (e.g., electroconvulsive therapy) are considered (Lee et al., 2012; Delaloye and Holtzheimer, 2014; Drobisz and Damborská, 2019; Lv et al., 2019; McAllister-Williams et al., 2020). Although significant gains have been made toward effective depressive disorder interventions, the increased challenge of treating depression with comorbidities (e.g., anxiety and chronic conditions) and the significant number of individuals who do not respond to intervention continue to underscore the complexity of the disorder and further need to better understand its etiology (Evans et al., 2005; Pollack, 2005; Center for Behavioral Health Statistics and Quality, 2018).

Anxiety is treated similarly to depression through psychotherapy (e.g., cognitive behavioral therapy) and medication. Due to the high co-morbidity of depression and anxiety, similar types of pharmacology are often used with the addition of benzodiazepines specifically for anxiety. Additional interventions such as certain types of biofeedback and mindfulness-based interventions have also shown promising results for some individuals with anxiety disorders (Rodrigues et al., 2017; Singh and Gorey, 2018; Tolin et al., 2020). Overall, treatments significantly improve the quality of life for most people with anxiety, but if terminated symptoms often return (Bandelow et al., 2017).

### 2.4 The etiologies of depression and anxiety are proposed to be multifactorial

The heterogeneous hypotheses for the development of depression have evolved over time (Shadrina et al., 2018). Today, genetic, biological, environmental, and psychological factors are proposed to contribute to the onset of depression (Sullivan et al., 2000; Krishnan and Nestler, 2008; López-León et al., 2008; Kendler and Gardner, 2016). Anxiety is also considered to have a complex etiology (Bandelow et al., 2017; Thibaut, 2017; Meier and Deckert, 2019). Neuroimaging is emerging as a method to identify depression and related subtypes (Dunlop and Mayberg, 2017; Peng and Yao, 2019). Although initial studies found differences between individuals with depression and controls (Kaiser et al., 2015; Kambeitz et al., 2017; Zhang et al., 2018, 2019) and identified potential predictors of response to intervention (Konarski et al., 2009; Siegle et al., 2012; Dunlop et al., 2017), the neuroimaging findings still need to be sufficiently replicated to have robust clinical implications.

Despite the well-documented physical, social, medical, and economic significance of depression and anxiety, there continues to be limited knowledge of their underlying mechanisms and causes. Further research is needed to better understand depression and anxiety, identify targeted interventions to more effectively manage it, with or without co-occurring conditions.

## 3 Assessing Depression- and Anxiety-Like Behaviors in NHPS

### 3.1 NHP literature regarding depression- and anxiety-like behaviors focuses mainly on marmosets and macaques

For this report, we searched PubMed up to February 2022 to find articles describing depression and/or anxiety and evaluation methods in NHPs. Keywords included non-human primate, monkey, marmoset, macaques, prosimian, ape, depression, anxiety, behavior, evaluation, MRI, PET, fMRI, DTI, and SPEC combined with peer-reviewed and English language as filters. Review articles were considered in order to condense multiple previous studies and relevant reference lists were reviewed to gather additional articles. Title and abstracts were screened for topic relevance. Articles were removed based on duplicative findings or not being aligned with the manuscript’s purpose. Fifty articles were selected and analyzed by research team members to extract information on models and tests applied per species and developmental stage. These articles are referenced in Tables s 2–4 describing methods of evaluation of depressive and anxious behaviors in species of marmosets and macaques.

**Table 2:** Selected peer-reviewed publications describing methods of evaluation of depression- and anxiety-like behaviors in species of marmosets (infant = 0–0.5 y; juvenile = 0.5–2 y; adult = 2–8 y).

**Reference**	**Age, Sex**	**Testing paradigm**	**Behavioral measures**	**Biological measures**	**Imaging**
Black-tufted Marmoset (*Callithrix penicillata*)					
Barros et al. (2000)	Adults, f & m	Predator test, diazepam dosing	Approach, lick, scent mark, scratch, locomotion	Effect of diazepam	None
Common Marmoset (*Callithrix jacchus*)					
Bassett et al. (2003)	Adults, f & m	Disruptive handling during care	Scratch	Urinary cortisol (μg/mg Cr/ml)	None
Pryce et al. (2004a,b)	Infants and juveniles, f & m	Maternal and social separation	Activity, social contact, vocalization	Urinary NE, ng/mg Cr/ml, systolic and diastolic pressure, and heart rate	None
Yokoyama et al. (2013)	Adults, m	Aggressive encounter trial	Approach contact, attack, confront, scent mark, sniff, locomotion	None	MRI, [11C]DASB-PET, FDG-PET
Mikheenko et al. (2015)	Adults, f & m	Human intruder, predator test	Distance to front of cage, locomotion, head-body bob, look, head-cock, vocalizations	Amygdala microdialysis for serotonin, systolic and diastolic pressure, and heart rate	MRI
Ash and Buchanan-Smith (2016)	Adults, f & m	Novel object or person	Latency to take rewards	None	None
Galvão-Coelho et al. (2017)	Juveniles, f & m	Social isolation	Observation of behavior	Fecal cortisol	None
Ishikawa et al. (2017)	Adults, m	Sleep/activity patterns by telemetry	Locomotion	Temperature, ECG, EMG, EOC	None
French (2019)	Infants, juveniles, adults, f & m	Predator test, human intruder, short-term social isolation (partner or infant), dyad partner-stranger choice test, open field novel object test, light-dark maze	Vocalization, avoidance or approach locomotion, reward learning	Urinary NE, ng/mg Cr/ml, systolic and diastolic pressure, and heart rate, oxytocin	High-resolution MRI, fMRI, two-photon laser capture microscopy
Ash et al. (2020)	Infants and juveniles, f & m	Behavior, cognitive and learning tests, family style (male parent highly involved vs. detached)	Observation of behavior, cognitive, and learning performance	None	None
Quah et al. (2020a)	Adults, f & m	Human intruder, predator test	Distance to front of cage, locomotion, head-body bob, head-cock, vocalizations	None	None
Quah et al. (2020b)	Adults, f & m	Human intruder, intramygdalar citalopram infusion	Distance to front of cage, locomotion, head-body bob, head-cock, vocalizations	Blood pressure	None

Abbreviations: y, years; [11C]DASB, 11C-3-amino-4-2-dimethylaminomethylphenylsulfanyl-benzonitrile (for serotonin activity); Cr, creatinine; ECG, electrocardiogram; EMG, electromyography; EOG; electrooculography; f, female; FDG, 18F-fluorodeoxyglucose (for glucose activity); fMRI, functional magnetic resonance imaging; kg, kilogram; m, male; mg, milligram; ml, milliliter; MRI, magnetic resonance imaging; NE, norepinephrine; ng, nanogram; PET, positron emission tomography; μg, microgram.

**Table 3:** Selected peer-reviewed publications describing methods of evaluation of depression- and anxiety-like behaviors in rhesus macaques (infant = 0–1 y; juvenile = 1–4 y; adult = 4–25 y).

**Reference**	**Age, Sex**	**Testing paradigm**	**Behavioral measures**	**Biological measures**	**Imaging**
Rhesus Macaque (*Macaca mulatta*)					
Harlow and Suomi (1971)	Infants and juveniles, f & m	Social isolation	Self-clasp, huddle, locomotion, exploration	None	None
Suomi et al. (1975)	Adults, f & m	New groups of friends, friends, and strangers, or isolation	Self-groom, self-clasp, huddle, stereotypy	None	None
Amaral (2002)	Adults, m	Novel object, bilateral lesions to amygdala	Latency to take reward near novel object	None	None
Strome et al. (2002)	Juveniles, f & m	Isolation *vs*. social context, intraventricular CRF infusion	Huddle, face to wall, self-clasp, locomotion, exploration	Glucose metabolism in infundibulum, amygdala, and hippocampus	MRI, FDG-PET
Kalin and Shelton (2003)	Juveniles, f & m	Human intruder test, serotonin-transporter polymorphism, lesions to Ce, intraventricular CRF infusion	Freeze, coo vocalization	CSF and plasma cortisol μg/dl, glucose metabolism in infundibulum, amygdala, and hippocampus	MRI, FDG-PET
Erickson et al. (2005)	Infants, f & m	Maternal separation	Social behavior, grooming	Plasma cortisol, CSF metabolites serotonin, dopamine, NE	None
Kalin et al. (2005)	Juveniles, m	Human intruder test	Freeze	None	MRI, FDG-PET
Winslow (2005)	Infants, f & m	Maternal separation	Social deficits, vocalizations, stereotypy	Oxytocin, AVP	None
O’Connor and Cameron (2006)	Infants, f & m	Human intruder test, play test, maternal separation	Social contact, play, movement, vigilance, vocalizing, stereotypy	None	None
Kalin et al. (2007)	Juveniles, f & m	Human intruder test, predator test, lesion to orbitofrontal cortex	Locomotion, freeze, coo, bark, aggression to experimenter	EEG, plasma and CSF cortisol (μg/dl, Acth, crf (pg/ml)	None
Spinelli et al. (2012)	Infants, f & m	Chronic maternal separation	Self-directed behaviors, withdrawal, locomotion, vocalizations, exploration	None	None
Rogers et al. (2013)	Juveniles, f & m	Human intruder test	Locomotion, freeze, vocalizations	CRHR1 genotypes	MRI, FDG-PET
Qin et al. (2015)	Adults, f	Subordinate status in social group	Huddling, lack of responsiveness	Hair cortisol	SPECT-rCBF
Kalin et al. (2016)	Juveniles, f & m	Human intruder test, CRF infusion	Freezing, cooing	Plasma cortisol	MRI, FDG-PET, fMRI
Zhang et al. (2016)	Juveniles, m	Early rearing adversity and later chronic stress (single housing with either space restriction, intimidation, long illumination, or fasting)	Huddling, decreased locomotion, decreased sexual behavior, repetitive stereotypies	Hair cortisol	None
Fox et al. (2019)	Juveniles, m	Human intruder test, over-expression of NTF3 in Ce and basal nucleus	Locomotion, freeze, vocalizations	RNA seq	MRI, FDG-PET
Kenwood and Kalin (2021)	Infants, juveniles and adults, f & m	Human intruder test, predator test, social behavior, lesions to Ce-region	Vocalization, freezing	CSF, plasma, and cortisol	MRI, FDG-PET
Villard et al. (2021)	Juveniles, m	Behaviorally inhibited (defined by BioBehavioral Assessment program) vs. randomly assigned age-matched control monkeys	None after the initial assessment	Histological analyses (volume and neuron number of hippocampus and amygdala	None
Wood et al. (2021)	Infants- sub-adult, f & m	6 month behavior during acute and chronic separation from mother, 4 years human intruder test, random assignment to adoptive or birth mother	Inactivity from social or environmental cues at infant separation test, intruder test aggression latency to approach, activity, exploration, social contact or withdrawal, stereotypy, and threat behaviors	Plasma ACTH	None

Abbreviations: y, years; ACTH, adrenocorticotropic hormone; AVP, vasopressin; Ce, central nucleus of the amygdala; CRF, corticotropin-releasing factor; CRHR1, CRH receptor 1gene; CSF, cerebral spinal fluid; dl, deciliter; EEG, electroencephalography; f, female; FDG, 18F-fluorodeoxyglucose (for glucose activity); fMRI, functional magnetic resonance imaging; m, male; MRI, magnetic resonance imaging; NE, norepinephrine; NTF3, neurotrophin-3 gene; PET, positron emission tomography; pg, picograms; rCBF, regional blood flow; RNA seq, RNA next generation sequencing; SPECT, single photon emission computed tomography.

**Table 4:** Selected peer-reviewed publications describing methods of evaluation of depression- and anxiety-like behaviors in cynomolgus macaques (infant = 0–1 y; juvenile = 1–4 y; adult = 4–25 y).

**Reference**	**Age, Sex**	**Testing paradigm**	**Behavioral measures**	**Biological measures**	**Imaging**
Cynomolgus Monkey (*Macaca fascicularis*)					
Shively et al. (2005)	Adults, f	Social subordinate stress, CRH injection, ACTH challenge, dexamethasone suppression	Alert, at rest, huddle, lack of responsiveness	Heart rate, mortality rate, blood ACTH and cortisol, metabolic and reproductive function	None
Shively and Willard (2012)	Adults, f	Subordination status in groups	Time in body contact, time alone, activity, huddle	Body weight, BMI, heart rate, lipid levels, cardiovascular disease risk	None
Willard et al. (2015)	Adults, f	Subordination status in groups, sertraline or placebo dosing	Scratch self-groom, slumped body posture, lack of responsiveness	Body weight, BMI, plasma sertraline, desmethylsertraline, CSF 5-HIAA	MRI
Xu et al. (2015)	Adults, f	Depressed compared to normal, ketamine dosing	Ingestion, thermo-regulatory, estrus, mating, resting, parental, grooming, embracing, conflict, vigilance, locomotive, verbal and non-verbal communication, and self-directed behaviors	GC/MS of 27 serum metabolites	None
Ishikawa et al. (2017)	Juveniles, m	Sleep/activity pattern by telemetry	Locomotor activity	ECG, EMG, EOC	None
Chu (2019)	Adults, f	Depressed (defined as huddling, low social contact, lack of motivation, and reduced alertness) compared to defined normal (no stereotypy), ketamine dosing	Passive state while sitting, reduced locomotion, tactile exploration, time on cage bars, nocturnal sleep	CSF levels of 5-HIAA, DA, HVA, NA	None
Qiu et al. (2019)	Juveniles, f & m	Wild type compared to BMAL1 knockout	Locomotor activity	EEG, Plasma levels of melatonin, testosterone, DHEA, cortisol	None
Qin et al. (2020)	Adults, f & m	Sleep/activity pattern by actigraphy vs. videography	Locomotor activity	None	None
Teng et al. (2021)	Juveniles, m	Chronic unpredictable mild stressors, noise, water deprivation, fasting, space restriction, cold stress, strobe light, foot shocks, human intruder test, out of reach reward test	Huddle, self-clasp, locomotion, lack of motivation for reward	Plasma μg/dl and hair cortisol, LC-MS analysis of 30 metabolites	None

Abbreviations: y, years; 5-HIAA, serotonin metabolite 5-hydroxyindole acetic acid; BMAL1, brain and muscle ARNT-like 1; BMI, body mass index; CRH, corticotrophin-releasing hormone; CSF, cerebral spinal fluid; DA, dopamine; DHEA, Dehydroepiandrosterone; ECG, electrocardiogram; EEG, electroencephalography; EMG, electromyography; EOG, electrooculography; f, female; GC-MS, gas chromatography-mass spectrometry; HVA, homovanillic acid; LC-MS, liquid chromatography-mass spectrometry; m, male; NA, noradrenaline.

Our literature search indicates that the most used NHP species in studies of depression and anxiety have been marmosets (genus *Callithrix*; Table 2) and macaques (genus *Macaca*; Tables s 3 and 4). Isolated publications describe methods to evaluate depressive and anxious behaviors for the gray mouse lemur, owl monkey, capuchin monkey, squirrel monkey, vervet monkey, bonnet monkey, Japanese macaque, olive baboon, and chimpanzee (Table 5). We did not delve deeper into the available literature on chimpanzees given the complicated ethics involved in performing great ape research and the severe restrictions the National Institutes of Health placed on research with this species in 2015.

**Table 5:** Selected peer-reviewed publications describing methods of evaluation of depression- and anxiety-like behaviors in lemur (infant = 0–0.2 y; juvenile = 0.2–1 y; adult = 1–12 y); owl monkey (infant = 0–0.5 y; juvenile = 0.5–2 y; adult = 2–12 y); capuchin (infant = 0–1 y; juvenile = 1–6y; adult = 6–25 y), squirrel monkey (infant = 0–1 y; juvenile = 1–4 y; adult = 4–20 y); vervet (infant = 0–1 y; juvenile = 1–3 y; adult = 3–12 y); baboon (infant = 0–1 y; juvenile = 1–4 y; adult = 4–25 y), chimpanzee (infant = 0–5 y; juvenile = 5–12 y; adult = 12–50 y).

**Reference**	**Age, Sex**	**Testing paradigm**	**Behavioral measures**	**Biological measures**	**Imaging**
Gray mouse lemur (*microcebus murinas*)					
Pifferi et al. (2019)	Adults, f & m	Open field, three-panel maze	Latency to move, avoidance locomotion	None	None
Fritz et al. (2020)	Adults, f & m	Open field with-without novel object, sequential choice test, light-dark maze	Exploration	None	MRI
Owl monkey (*Aotus nancymaae*)					
Osman et al. (2020)	Juveniles, f & m	Maternal separation	Pace, rest, head twirl	Hair cortisol (pg/ml), infection history	None
Capuchin (*Cebus apella)*					
Sorrentino et al. (2012)	Juveniles and adults, f & m	Observations in wild group	Scratch, aggressive and self-directed behaviors, vocalizations	None	None
Kean et al. (2017)	Juveniles and adults, f & m	Observations in wild and captive groups, food competition	Alarm call, scratch	Fecal cortisol	None
Squirrel monkey (*Saimiri sciureus)*					
Canon and Houser (1978)	Adults, f	Discrete trial conflict test with 5–7.5mg/kg chlordiazepoxide, 1–2mg/kg diazepam, 125–150 mg/kg meprobamate, 4 mg/kg chlorpromazine	Task performance in conflict approach-avoidance trials	None	None
Levine and Mody (2003)	Juveniles, f & m	Maternal separation	Vocalizations	Plasma and CSF cortisol (μg/dl), CSF monoamine metabolites MHPG, HVA, 5HIAA (ng/ml)	None
Vervet monkey (*Chlorocebus aethiops)*					
Marais et al. (2006)	Infants, f & m	Observations in captive groups	Stereotypy, huddling, exploration, activity	CSF cortisol (μg/dl), CSF monoamine metabolites MHPG, HVA, 5HIAA, DOPAC	None
McDougall (2011)	Adults, f	Observations in wild groups	Scratch, groom, self-touch, body shake, yawn	None	None
Bonnet Monkey (*Macaca radiata*)					
Jackowski et al. (2011)	Juveniles, m	VFD early stress paradigm as infants, human intruder test	Fearful responses	Genotype for “s” allele	MRI
Japanese Macaque (*Macaca fuscata*)					
Troisi et al. (1991)	Adults, f	Observation of maternal anxiety	Scratch	None	None
Olive baboon (*Papio anubis)*					
Castles et al. (1999)	Adults, f	Observations in wild groups	Scratch, groom, self-touch, body shake, yawn	None	None
Chimpanzee (*Pan troglodytes*)					
Baker and Aureli (1997)	Juveniles and adults, f & m	Observations in captive groups	Self-groom, scratch, yawn, social behavior, exploration, vocalization, locomotion	None	None

Abbreviations: y, years; 5-HIAA, 5-hydroxyindole acetic acid; AD, Alzheimer’s disease; CSF, cerebral spinal fluid; DOPAC, dopamine metabolite dihydroxyphenylacetic acid; f, female; HVA, catecholamine metabolite homovanillic acid; m, male; MHPG, norepinephrine metabolite 3-Methoxy-4-hydroxyphenylglycol; MRI, magnetic resonance imaging; VFD, variable foraging demand.

Both, marmoset and macaque monkeys share ~93% sequence identity with the human genome (Marmoset Genome and Analysis, 2014; Warren et al., 2020), yet they have critical species-specific differences to be considered for depression and anxiety studies. Marmosets are small-bodied (300–500 g in captivity), arboreal new world primates native to Brazil. The common marmoset (*Callithrix jacchus*) has been widely used to model and study depression and anxiety behaviors, compared to its close relative, the black-tufted marmoset (*Callithrix penicillata*). Among anthropoid primates, marmosets have a smaller, lissencephalic brain and are the shortest-lived (average 5–7 years, max 20) and the most fecund (twins born every 6 months). Marmosets have been used for many years in biomedical research, and within the last decade, their popularity increased dramatically, prompted to a large degree by their use in neuroscience research and their potential for developing germ-line genomic edited subjects (Rodriguez-Callejas et al., 2016; Servick, 2018). In contrast, macaques are old-world NHPs, native to Asia and North Africa. They are relatively large-bodied (2.4–5.5 kg, smaller species; 10–14 kg, larger species) and long-lived (>30 years), with a larger, more complex brain closer to humans. Macaques are the most commonly used NHPs in biomedical research and as such, it is not surprising that they are also the most common NHP used to model depression- and anxiety-like behaviors, specifically the rhesus monkey (*Macaca mulatta*) and cynomolgus monkey (*Macaca fascicularis*).

### 3.2 The methods for modeling and detecting depression- and anxiety-like behaviors in NHPs can produce controversial results

A notable finding during the analysis of the literature was that the majority of the NHP publications describe a mix of overlapping signs present in depression or anxiety, complicating the task of distinguishing between studies focused on either disorder, and yet underscoring the difficulty of their differential diagnoses in primates. This mixed reporting of general signs of depression or anxiety was observed in many of the identified marmoset studies, although new reports are emerging specific to depression- (Galvão-Coelho et al., 2017) and anxiety-like (Quah et al., 2020a,b) behaviors in this species (Table 2). In contrast, for macaques (Tables s 3 and 4) we were able to identify 10 macaques studies focused on depression (Harlow and Suomi, 1971; Suomi et al., 1975; Strome et al., 2002; Shively et al., 2005; Qin et al., 2015; Willard et al., 2015; Xu et al., 2015; Zhang et al., 2016; Chu, 2019; Teng et al., 2021), eight on anxious temperament (Amaral, 2002; Kalin and Shelton, 2003; Kalin et al., 2005, 2007, 2016; Rogers et al., 2013; Fox et al., 2019; Kenwood and Kalin, 2021), while the rest described general signs of either condition.

As we mentioned in the introduction, it is difficult to know whether an animal is truly depressed or anxious, yet some of its behaviors resemble the human condition (Bliss-Moreau and Rudebeck, 2021). The critical issue is how to generate a NHP model that reliably produces depression- and anxiety-like behaviors. NHPs may spontaneously present depression- and anxiety-like behaviors (Xu et al., 2015; Chu, 2019) but their detection depends on careful observation of a large population of animals. As a modeling alternative, disrupted husbandry or separation, either short or long-term, from either a parent or other social partner has been used to elicit a depressed- or anxious-like condition in both marmosets and macaques (Harlow and Suomi, 1971; Erickson et al., 2005; Winslow, 2005; O’Connor and Cameron, 2006; Spinelli et al., 2012; Zhang et al., 2016; French, 2019; Villard et al., 2021; Wood et al., 2021).

Genotyping has matched specific alleles with spontaneous expression of anxious temperament in macaques, and had helped identified carriers at risk for anxiety (Jackowski et al., 2011; Rogers et al., 2013). Intracerebral delivery of viral vectors encoding for those alleles is proposed as a shortcut to modeling genetic predisposition (Fox et al., 2019). Increased intracerebral corticotropin releasing factor (CRF; Strome et al., 2002; Kalin and Shelton, 2003; Shively et al., 2005) and lesions to the central nucleus of the amygdala or orbitofrontal cortex (Amaral, 2002; Kalin and Shelton, 2003; Kalin et al., 2007; Kenwood and Kalin, 2021) have also been applied to develop a depression- or anxiety-like phenotype.

Although some of these behaviors can present spontaneously, investigators have created testing paradigms to facilitate the detection by evaluating the subjects’ response to what appears to be an aggressive intruder (i.e., human intruder paradigm (Kalin and Shelton, 2003; Kalin et al., 2005, 2007, 2016; O’Connor and Cameron, 2006; Rogers et al., 2013; Mikheenko et al., 2015; French, 2019; Kenwood and Kalin, 2021); a predator (Barros et al., 2000; French, 2019; Kenwood and Kalin, 2021), or a novel object or person (Amaral, 2002; Ash and Buchanan-Smith, 2016). NHPs’ behavioral responses to these paradigms, such as freezing or vocalizations, are typical adaptive defensive behaviors, yet the evaluation does not rely on their presence or absence but rather on the characteristics of the response (e.g., intensity, duration), which in NHPs exhibiting depression- and anxious- like behaviors differ from normal parameters (Fox and Kalin, 2014).

Taken together, these established techniques provide a framework for the use of marmosets and macaques to model and detect depression- and anxiety-like behaviors.

### 3.3 Studying depression- and anxiety-like behaviors in NHPs require species- and age-specific evaluation

Marmosets and macaques share depression- and anxiety-like behaviors that are similar to human ones but also have species-specific characteristics that change with age. Identifying and scoring these behaviors are the main evaluation tool for studies of these disorders in NHPs (Table 1).

Marmosets’ depressive-like behaviors include less locomotion, increased contact (phee) calling, and/or longer latency to take rewards. Their anxiousness is expressed by avoidance, scent-marking, increased alarm (tsk) or aggressive (chatter, er-er) calls, excessive scratching, licking, and sniffing, or head cocking behavior. Measures of these behaviors need to consider that younger marmosets are more active, sociable, and playful compared to adult animals, who themselves, spend more time grooming and cuddling.

Depressive-like behaviors in macaques also include decreased locomotion, in addition to adopting a self-clasping, huddle posture, increased coo calls, and/or lack of responsiveness for social contact or rewards. Their anxious-like behaviors are freezing, the presence of alarm bark, or excessive scratching, pacing, self-grooming, and yawning. With respect to age, infant rhesus’ behaviors are particularly affected by contact and separation from caregivers, while juveniles are socially active and withdrawal from their group is indicative of possible distress. Adult rhesus are, in general, protectors of the group and affected by strangers, whereas older animals are comfort-seeking with less activity, pursuing contact and grooming.

Like in humans, specific cognitive and learning tests (that can also assess attention) can detect impaired performance in NHPs displaying depression- and anxiety-like behaviors (Sridharan et al., 2012; Ash et al., 2020; Nephew et al., 2020). For rhesus macaques, most of these tests are adaptations of human versions, such as the Wisconsin card sorting task (WCST) and conditioned reward learning (Sridharan et al., 2012). For marmosets, the testing paradigms are often based on rodents ones, like an open field, light-dark maze, classical conditioning, and reward learning (Ash and Buchanan-Smith, 2016; French, 2019; Ash et al., 2020).

### 3.4 Biological measures associated with depression- and anxiety-like behaviors in NHPs provide further translational insight

Routine clinical observations, including monitoring of food intake and body weight regularly performed in NHP colonies, can indicate loss of appetite and somatic signs associated with depression and anxiety. Although not described in reports studying these disorders, body mass index (BMI; Shively and Willard, 2012; Willard et al., 2015) or dual-energy x-ray absorptiometry (DXA; Yamada et al., 2013; Mattison et al., 2017; Kraynak et al., 2019) can be used in macaques and marmosets to measure body composition and further evaluate animals with loss of appetite manifested by a change in body weight and adiposity. Fitbit-like monitors can be used to assess activity levels and circadian rhythm (Oler et al., 2010; Yamada et al., 2013, 2018; Qiu et al., 2019) and identify sleep disturbance as manifested by increased overnight activity.

Measures of heart rate, blood pressure, and sleep using implanted telemetry and measurements of circulating levels of cortisol, norepinephrine, and serotonin, from plasma, urinary or fecal samples (Bassett et al., 2003; Kalin and Shelton, 2003; Pryce et al., 2004a,b; Erickson et al., 2005; Qin et al., 2015; Kalin et al., 2016; Qiu et al., 2019) along with additional hormone and neurotransmitter metabolites from cerebrospinal fluid (Erickson et al., 2005; Winslow, 2005; Kalin et al., 2007; Mikheenko et al., 2015; Willard et al., 2015; Chu, 2019; French, 2019; Teng et al., 2021) are used to measure physiological responses that correlate with depression and anxiety behaviors. Increased heart rate, blood pressure, cortisol, NE, and serotonin metabolites are often correlated with these behaviors.

Modification of human brain imaging techniques to NHPs is part of the assessment battery in depression and anxiety paradigms (Strome et al., 2002; Kalin and Shelton, 2003; Kalin et al., 2005, 2016; Jackowski et al., 2011; Rogers et al., 2013; Yokoyama et al., 2013; Mikheenko et al., 2015; Qin et al., 2015; Fox et al., 2019; French, 2019; Kenwood and Kalin, 2021). In addition to structural differences with magnetic resonance imaging (MRI) and white matter connections using diffusion tensor imaging, brain activity can be evaluated by functional MRI that utilizes blood oxygen levels to assess neuron activity (Vanduffel et al., 2014), positron emission tomography (PET) with F^18^ Fluorodeoxiglucose to assess brain consumption of glucose (Strome et al., 2002; Kalin and Shelton, 2003; Rogers et al., 2013; Yokoyama et al., 2013; Kalin et al., 2016; Fox et al., 2019; Kenwood and Kalin, 2021) or single photon emission computed tomography (SPECT) with regional blood flow (rCBF; Qin et al., 2015). PET with C^11^ 3-amino-4-(2-dimethylamino methylphenylsulfanyl)-benzonitrile can be used to assess serotonin pathways in the brain by measuring binding to the serotonin transporter (Yokoyama et al., 2013).

## 4 Gaps in Knowledge, Ethical Considerations, and Alternatives for Studying Depression and Anxiety in NHPS

### 4.1 Analysis of depression- and anxiety-like behaviors in NHPs across the lifespan

Our analysis was able to identify relevant information for both males and females in multiple species at different developmental timeframes including infant, juvenile, and an adult but not aged NHPs. In humans, the prevalence of depression differs with age. Given that NHP behavior varies across age stages, it is critical to adapt these tests to evaluate age-appropriate activities and responses. Moreover, these behaviors can be identified as comorbid conditions along with various age-related chronic diseases such as diabetes, arthritis, stroke, and PD. The lack of reports regarding depression- and anxiety-like behaviors in older NHPs represents a significant gap in the literature that stymies the use of these highly translational models in addressing important biomedical research questions.

### 4.2 Depression- and anxiety-like behaviors as a comorbidity of NHP models of human disease and a target for precision medicine

Similar to aging models, research on depression- and anxiety-like behaviors as a comorbidity in NHP models of disease has not been prioritized and is urgently needed in order to help affected patients. These types of studies also provide an opportunity to consider depression and anxiety from a different perspective, unravel pathological mechanisms, and identify novel therapeutic approaches. Two notable examples to consider are NHP models of PD and of interferon alpha (IFN-α)-treatment.

It is estimated that 30%–40% of PD patients present with depression and only 20% are treated. Oral dopamine replacement with L-DOPA (a dopamine precursor), the mainstay therapy for PD, is marginally effective against depression and anxiety (Frisina et al., 2009; Huot et al., 2017). In addition to dopaminergic loss, PD neurodegeneration also affects other neurotransmitters, including norepinephrine and serotonin, which are proposed to contribute to the depression and anxiety of PD (Draoui et al., 2020; Mendonça et al., 2020). Interestingly, monkey models of PD induced by the neurotoxin MPTP also present significant dopaminergic loss as well as loss of norepinephrinergic and serotoninergic innervation (Masilamoni and Smith, 2018). Underdiagnoses of depression and anxiety are common, as frequent indicators of underlying depression can be obscured by some PD symptoms, such as sleep disturbances and slowness of movement. Although depression in rodent models of PD has been widely studied (Schintu et al., 2012; Hussein et al., 2021), reports of depression and anxiety in NHP PD models are missing, which has affected progress in identifying biomarkers and treatments. Anxious pacing has been described in transgenic rhesus macaques overexpressing A53T mutated alpha-synuclein (Niu et al., 2015). Our group has recently identified depression and anxiety behaviors in rhesus macaques with neurotoxin-induced hemiparkinsonism. In addition to typical PD motor signs, the animals presented a lack of interest, anxious pacing, and excessive grooming, indicating a combination of depression- and anxiety-like signs. These behaviors improved after intrastriatal grafting of autologous (from the same animal) induced pluripotent stem cell-derived autologous dopaminergic neurons (Tao et al., 2021). This unexpected finding suggests that, unlike oral dopamine replacement, targeted and localized intracerebral dopamine may be beneficial against depression and anxiety in PD.

Interferon alpha (IFN-α), an inflammatory cytokine, has been successfully used as immunotherapy for patients with chronic hepatitis B and C, and certain cancers, like malignant melanoma and Karposi sarcoma (Pestka et al., 2004). Yet its administration can induce numerous side effects, including depression and anxiety (Sleijfer et al., 2005; Pinto and Andrade, 2016). IFN-α dosing in rhesus macaques also induced depression- and anxiety-like behaviors, identified as increased huddling, self-scratching, body shakes, and yawning. These behaviors were associated with increased ACTH levels in plasma and lower homovanillic acid (a dopamine metabolite) levels in CSF (Felger et al., 2007). Follow up NHP studies demonstrated that IFN-α reduces the striatal availability of dopamine precursors that can be reversed by L-DOPA administration. This research has led to a number of clinical studies testing DA replacement in depression associated with high levels of inflammatory cytokines (Escalona and Fawcett, 2017; Rutherford et al., 2019).

The information gathered from seemingly two different lines of research, PD, and IFN-α, provides new insight in the role of dopaminergic neurotransmission and inflammation in depression and anxiety. It also emphasizes the need to develop precision medicine approaches to optimize treatments depending on the underlying condition associated with depression and anxiety that can be further facilitated by studies in a new generation of NHP models of disease based on germline (Aida and Feng, 2020) or somatic cell genomic modification (Saha et al., 2021).

### 4.3 Maximizing the usefulness of collected outcome measures as part of an ethical approach to NHP research

The welfare of animals used in biomedical research is extremely important both ethically and because well cared for animals make the best research subjects. Worldwide, consideration of the use of animals in research is based on the principle of the three R’s; replacement, reduction, and refinement (Russel and Burch, 1959). These principles guide researchers to use non-animal alternatives when possible and if not possible to use lower phylogenetic species of animals, use the fewest animals necessary to appropriately address the research question, refine techniques to minimize animal suffering, and use assessments tools that are most efficient at collecting the necessary data.

A number of established and validated techniques can be applied to assess NHPs’ depression- and anxiety-like behaviors, which were not specifically designed to assess these disorders, but can be easily adapted for such use. As we mentioned above, routine clinical observations performed in NHP colonies are great resources for assessing loss of appetite and somatic signs often associated with depression. The use of fitbit-like monitors is another non-invasive, easy approach to record activity levels and evaluate circadian rhythm and sleep patterns (Yamada et al., 2013, 2018; Kraynak et al., 2019; Qin et al., 2020). Traditional cognitive testing in macaques has relied on the Wisconsin general testing apparatus (WGTA) and conditioned reward learning (Sridharan et al., 2012). These tests require devoted, highly trained personnel and extensive training for the animals that can last many months, yet some animals even without depression and anxiety, are still nonreliable or nonperformers. Alternative cognitive testing such as the use of a multilevel puzzle feeder task that the animals can perform in their home cage, can begin to address these challenges (Watson et al., 1999).

Behavioral data can be collected either in person or by scoring videos, using focal animal sampling, all occurrence sampling, or instantaneous scan sampling. In-person scoring can be expeditious and provide greater insight into the animal’s overall demeanor, but it can be heavily influenced by the scorer. To minimize data variability, it is critical that beyond blind evaluation, the investigators are well-known by the animals, trained for reliability, and have time to dedicate to the testing. Scoring of videorecorded sessions can be done anytime, off-site, and inter-rater-reliability tests can be easily performed. Yet, good quality recordings require animals’ habituation to the set-up and personnel, plus the application of systematic methods by blind investigators. The behavioral data can be used to assess animals for motivation and interest levels, locomotor patterns, social interactions, anxious-like behavior, apparent sadness, and any stereotypical behaviors (Harlow and Suomi, 1971; Suomi et al., 1975; Troisi et al., 1991; Barros et al., 2000; Strome et al., 2002; Erickson et al., 2005; Winslow, 2005; O’Connor and Cameron, 2006; Shively et al., 2009; Shively and Willard, 2012; Spinelli et al., 2012; Willette et al., 2012; Rogers et al., 2013; Qin et al., 2015; Kalin et al., 2016; Ash et al., 2020; Kenwood and Kalin, 2021).

The scientific value and utility of NHP models for the study of depression and anxiety are mentioned above, but the ethical decision to use NHP models requires a somewhat different calculus. These decisions must be based on consideration of a harm-benefit analysis rooted in scientifically valid information. Specifically, regarding whether the benefit gained from using the model outweighs any potential harm to the animals. For example, for some forms of depression and anxiety research one should ask, does the information garnered using a stress-induced model outweigh the level of harm experienced by the animals in the study? Can spontaneous behaviors provide enough insight in the condition? Could a terminal analysis of the brain be replaced by an *in vivo* imaging study? The inverse must be considered as well. Does the potential harm in lack of knowledge outweigh the benefit to the animal in not being used in research? For example, without the use of animal research will we fail to identify effective new therapies for depression and anxiety, or will the lack of animal studies lead to an ineffective or dangerous treatment being employed? It is our obligation as researchers to use animal models respectfully and appropriately. This includes using the most appropriate model to answer our questions, inducing the minimal amount of stress possible to achieve the aims of the study, using the fewest animals possible while maintaining an appropriately powered study, and to reduce the harm to each individual through the use of refinements whenever possible.

## 5 Conclusion and Future Directions

Depression and anxiety affect millions of people worldwide across the lifespan. NHP research is providing clues on pathophysiology, diagnoses, and treatments for these disorders, but much work remains to be done. The gaps in knowledge on depression and anxiety with aging and comorbidities are opportunities to advance the field, ethically leveraging the resources already generated. These emerging datasets in NHPs can be the canvas for the application of novel targeted testing approaches (e.g.,: genetic manipulation, imaging in awake behaving animals) to help identify mechanisms, create precise interventions and improve treatment outcomes. They will require a team-oriented approach combining disease-specific experts and NHP researchers in order to create encompassing experimental designs, maximize results, and ultimately help patients affected by these disorders.

## Author Contributions

KA provided insight on the human condition. RC and NS-D contributed expertise in nonhuman primate evaluations and interpretation. SK and NS-D critically contributed to the creation of Tables s 2–5. ME conceived the idea for the manuscript and provided neurobiological basis. All authors contributed to the article and approved the submitted version.
